# Supplementary material for: Chemokines modulate glycan binding and the immunoregulatory activity of galectins
Source: Commun Biol. 2021 Dec 20;4:1415. doi: 10.1038/s42003-021-02922-4 (PMC8688422; doi:10.1038/s42003-021-02922-4)
Supplement: Supplementary file 2 — Supplementary Information [file 42003_2021_2922_MOESM2_ESM.pdf]

Supplementary Figures to:

Chemokines modulate glycan binding and the immunoregulatory activity of galectins.

Lucía Sanjurjo, Iris A. Schulkens, Pauline Touarin, Roy Heusschen, Ed Aanhane, Kitty C.M. Castricum, Tanja D. De Gruijl, Ulf J. Nilsson, Hakon Leffler, Arjan W. Griffioen, Latifa Elantak, Rory R. Koenen, Victor L.J.L. Thijssen

Supplementary figure 1. Specific protein-protein interactions occur between galectin-1 and different cytokines/chemokines.

Galectin-1

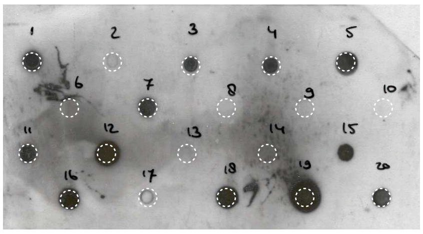

1. Anti gal-1 mab
2. Anti ICAM1 mab
3. Anginex
4. Anginex
5. bpep28
6. BSA
7. IL1a
8. IL4
9. IL6
10. IL8
11. IL17a
12. CCL5
13. IFNg
14. TNFa
15. CXCL4
16. CXCL4
17. CXCL4
18. CXCL4
19. Gal-1
20. Gal-9

Galectin-9

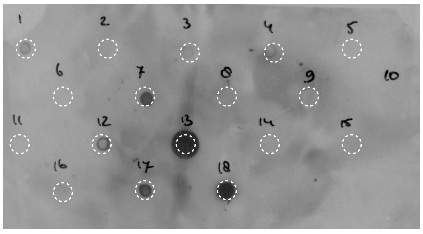

1. anti gal-1 mab
2. anti ICAM1 mab
3. anti gal-1 pab
4. Anginex
5. bpep28
6. BSA
7. IL1a
8. IL4
9. IL6
10. IL8
11. IL10
12. IL17a
13. CCL5
14. IFNg
15. TNFa
16. CXCL4
17. rGal-1
18. rGal-9

No galectin control

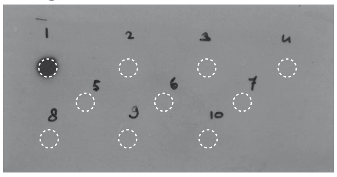

1. Positive control
2. BSA
3. Anginex
4. IL1a
5. IL17a
6. CCL5
7. CXCL4
8. IL8
9. FLNA
10. MT2A

Spot blot analysis. The listed proteins were spotted onto nitrocellulose filter and following incubation with galectin-1 (left panel) or galectin-9 (middle panel), protein-protein interactions were revealed by staining with anti-galectin-1 antibody (left panel) or anti-galectin-9 antibody (middle panel). Bovine serum albumin (BSA) and anti-ICAM monoclonal antibody served as negative control. In addition, ommission of incubation with gal-1 was used to exclude direct binding of galectin-1 antibody to cytokines (right panel). Of note, occassionally some weak background signal was observed, e.g., mAb gal-1/gal-9, mAb ICAM-1/gal-1, IL17a/gal-9. This was probably due to excess protein blotting and multiple antibody incubation steps, and therefore, these low signals were not considered as true interactions.

Supplementary figure 2. Surface plasmon resonance (Biacore) analysis.

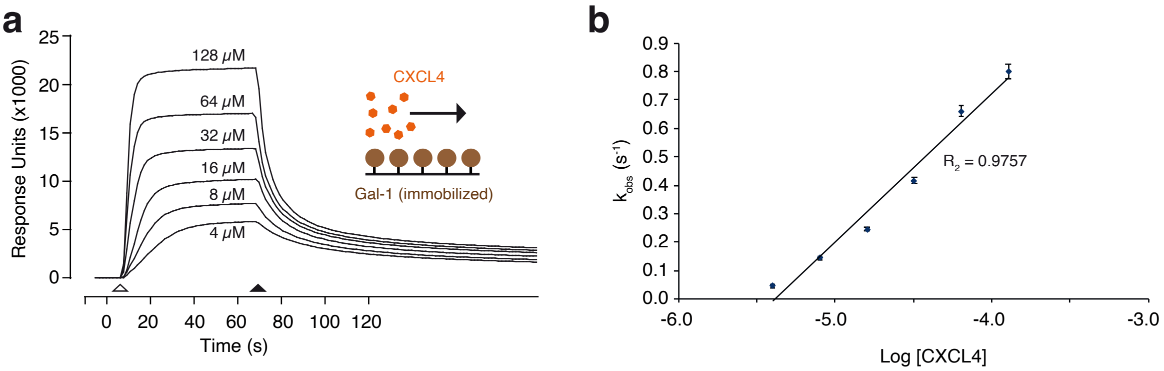

a Surface plasmon resonance analysis of interaction between CXCL4 and galectin-1. Increasing amounts of CXCL4 were run over the chip with immobilized galectin-1, resulting in an increase in response units (RU). **b** The observed association rates (k_{obs}) plotted as a function of analyte concentration with a slope equal to the association rate constant (k_a).

Supplementary figure 3. Peak intensity ratio analysis of ^{15}N amide probes of galectin-1.

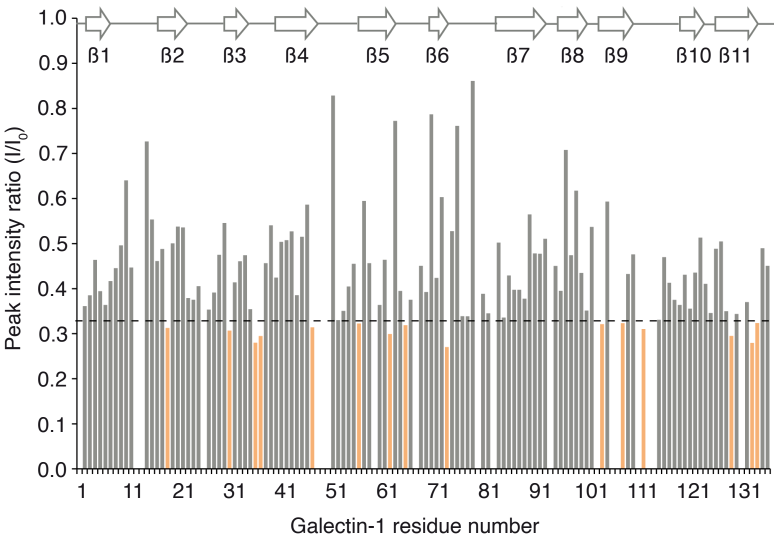

Normalized peak intensity ratio analysis (I/I_0) of galectin-1 bound to CXCL4. Dotted lines represent 1σ from the average I/I_0. Ratios below the threshold are colored in light orange.

Supplementary Figure 4. Galectin-1 interaction to the CXCL4 ß-sheet region (CXCL4$^{22\text{-}54}$).

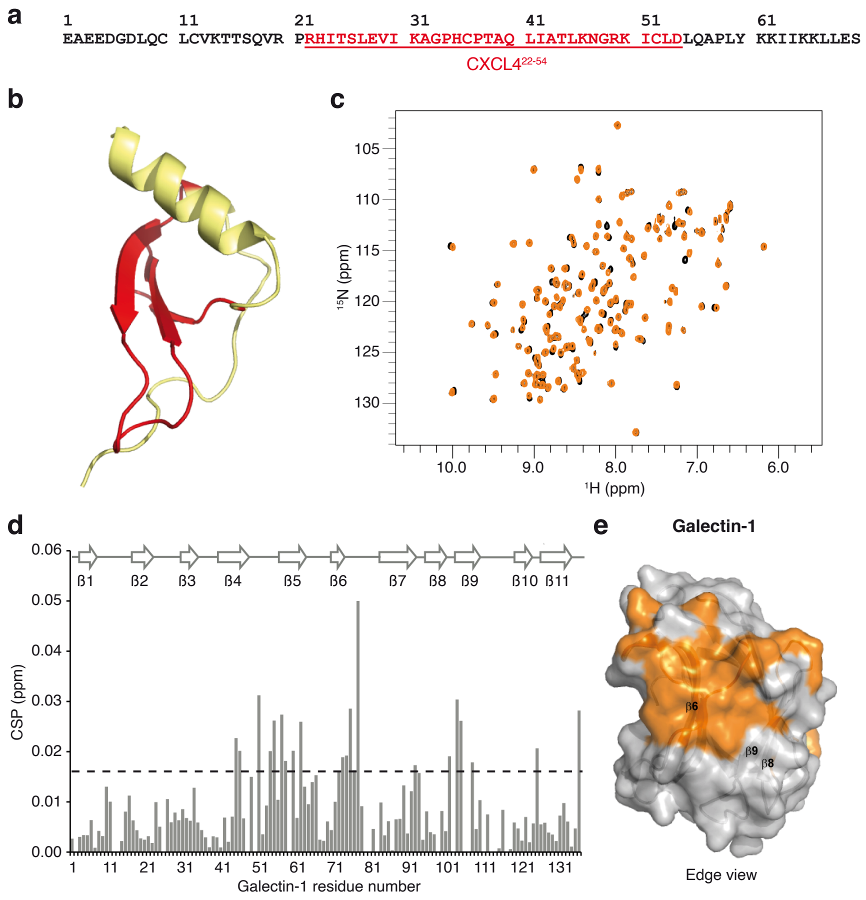

a CXCL4 amino acid sequence with the sequence of the b-sheet used for galectin-1 interaction highlighted in red. **b** Structure of the CXCL4 protein with the beta-sheet region used for galectin-1 interaction colored red. **c** Overlay of ^{1}H-^{15}N HSQC spectra of ^{15}N-labeled galectin-1 free (black trace) and bound to CXCL4$^{22\text{-}54}$ (orange trace). **d** Histogram plot of CSPs observed for each galectin-1 resonance upon interaction to CXCL4$^{22\text{-}54}$. The dashed line represents 1s from the average CSP, thereby defining the threshold selection for the most affected residues. Galectin-1 secondary structures are depicted above the plot. **e** Chemical shift perturbation mapping onto galectin-1 monomer structure which is shown as a semitransparent solvent-accessible surface with a ribbon model displayed below the surface. Residues presenting CSPs above the threshold (defined in (b)) are colored in orange. Main beta-strands affected are labeled.

Supplementary Figure 5. CXCL4 specifically affect glycan-binding affinity of galectin-1.

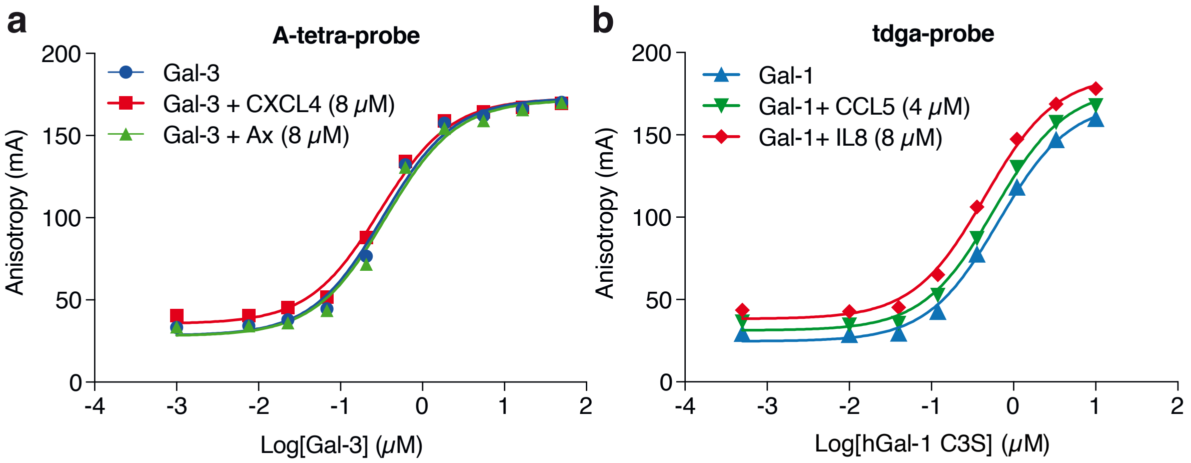

a Anisotropy analyses using a fluorescently labeled high affinity A-tetra-probe with increasing galectin-3 concentrations in the absence (blue line) of cytokine or in the presence of either CXCL4 (red line) or anginex (green line). No shift in anisotropy is observed. N=1. **b** Anisotropy analyses using a fluorescently labeled high affinity tdga-probe with increasing galectin-1 concentrations in the absence of cytokine (blue line) or in the presence of either CCL5 (green line) or IL8 (red line). N=1

Supplementary Figure 6. Time- and concentration-dependent induction of Jurkat apoptosis by galectins.

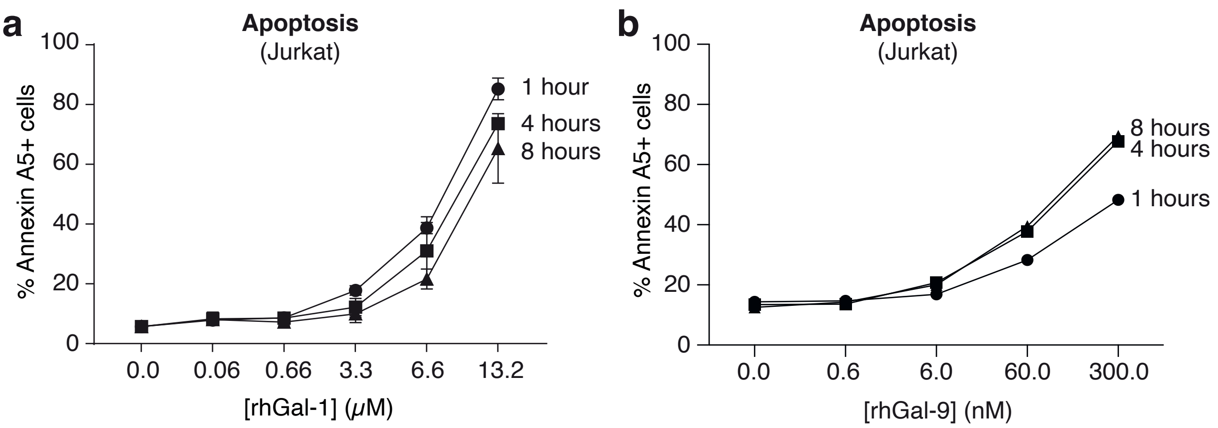

Jurkat cells were incubated during the indicated times and doses with galectin-1 (**a**) or galectin-9 (**b**). Apoptosis was assessed by annexin A5 and PI staining and FACS analysis. Graphs show data from at least 3 independent experiments.

Supplementary Figure 7. Analysis of concentration-dependent immune cell activation by PHA-L treatment.

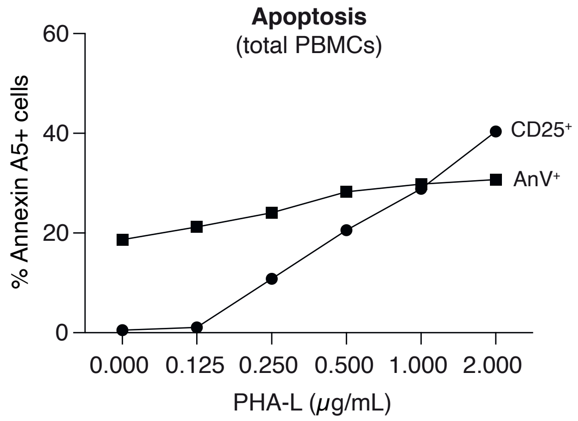

PBMCs were treated for 24 hours with phytohemagglutinin-L (PHA-L) at the indicated concentrations. Apoptosis was assessed by FACS using annexin A5. Cell activation was assessed by FACS using CD25 staining. N=1.

Supplementary Figure 8. Chemokines do not affect apoptosis of resting and activated PBMCs.

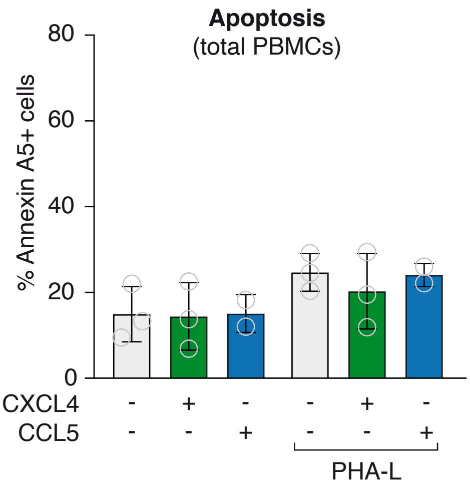

Apoptosis as determined by FACS analysis (Annexin A5$^+$ and PI$^+$ staining). Both resting and activated peripheral blood mononuclear cells (PBMCs) were incubated for 24 hours with 12 µM CXCL4 or 36 nM CCL5. PBMC-activation was induced by 24 hours treatment with 1 µg/mL phytohemagglutinin-L (PHA-L) prior to treatment with chemokines. N=2/3 independent experiments.

Supplementary Figure 9. FACS gating strategy for PBMC sub-populations.

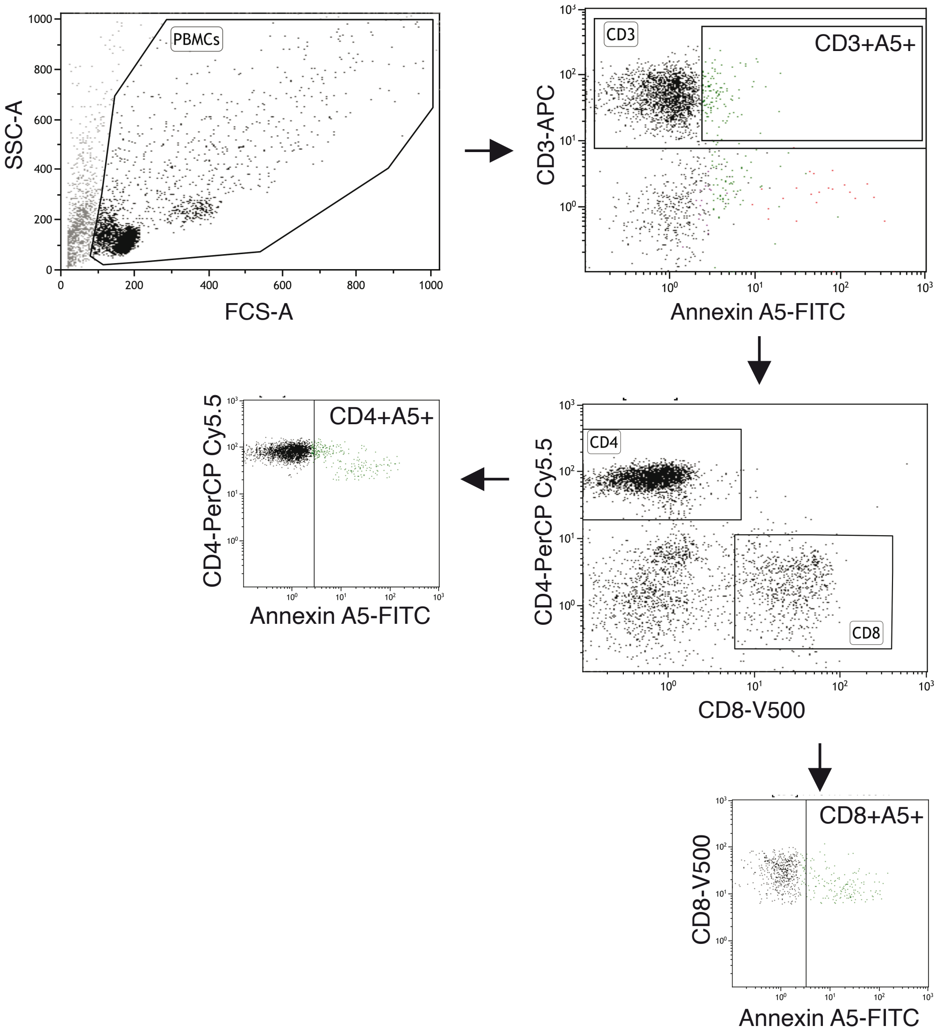

The gating strategy used to evaluate by flow cytometry the different circulating PBMC subsets based on the expression of CD3, CD4 and CD8 markers is shown. Forward and side scatter was used to exclude cell debris. Anti-CD3-APC was used to define total T cells that were further subdivided into CD4+ T Cells and CD8+ T cells based on staining with anti-CD4-PerCP Cy5.5 and anti-CD8-V500. Annexin A5-FITC was used to measure the apoptotic status of different cell subsets.

Supplementary Figure 10. Effect of galectin-cytokine interactions on apoptosis of CD14+ cells.

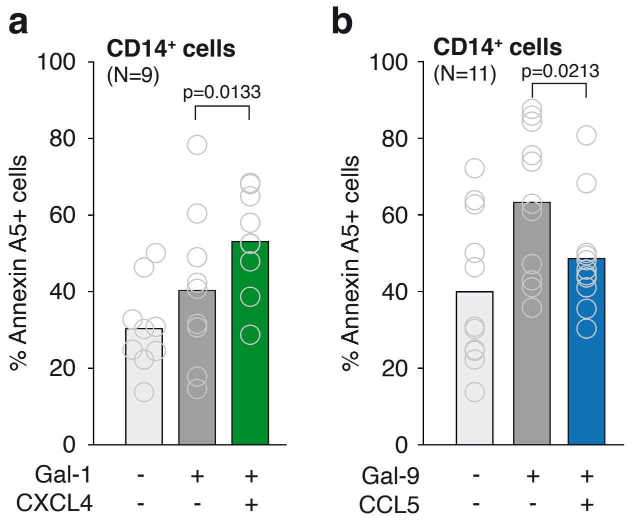

Apoptosis of CD3⁻/CD14⁺ PBMCs, as determined by FACS analysis (Annexin A5⁺ and PI⁺ staining) in response to treatment similar as in Figure 5.
