# Supplementary material for: Chemokines modulate glycan binding and the immunoregulatory activity of galectins
Source: Commun Biol. 2021 Dec 20;4:1415. doi: 10.1038/s42003-021-02922-4 (PMC8688422; doi:10.1038/s42003-021-02922-4)
Supplement: Supplementary file 3 — Description of Additional Supplementary Files [file 42003_2021_2922_MOESM3_ESM.pdf]

Description of Additional Supplementary Files

File name: Supplementary Data.
Description: Original scans of the Western blots shown in Figure 1 as well as the source data presented in Figures 3-5.
